# Workflow Analysis for CGH Generation with Speckle Reduction and Occlusion Culling Using GPU Acceleration

**DOI:** 10.3390/s25206492

**Published:** 2025-10-21

**Authors:** Francisco J. Serón, Alfonso Blesa, Diego Sanz

**Affiliations:** 1 Department of Computer Science, Universidad de Zaragoza, Escuela de Ingeniería y Arquitectura—EINA, 50008 Zaragoza, Spain; seron@unizar.es; 2 Department of Electronics and Communication, Universidad de Zaragoza, Escuela Universitaria Politénica de Teruel—EUPT, 44003 Teruel, Spain; 3 Department of Computer Science, Universidad de Zaragoza, Escuela Universitaria Politénica de Teruel—EUPT, 44003 Teruel, Spain

**Keywords:** CGH, speckle denoising, occlusion culling, point-cloud method, ray tracing, CGH simulation

## Abstract

Although GPUs are widely used in Computer-Generated Holography (CGH), their specific application to concrete problems such as occlusion or speckle filtering through temporal multiplexing is not yet standardized and has not been fully explored. This work aims to optimize the software architecture by taking the GPU architecture into account in a novel way for these particular tasks. We present an optimized algorithm for CGH computation that provides a joint solution to the problems of speckle noise and occlusion. The workflow includes the generation and illumination of a 3D scene, the calculation of the CGH including color, occlusion, and temporal speckle-noise filtering, followed by scene reconstruction through both simulation and experimental methods. The research focuses on implementing a temporal multiplexing technique that simultaneously performs speckle denoising and occlusion culling for point clouds, evaluating two types of occlusion that differ in whether the occlusion effect dominates over the depth effect in a scene stored in a CGH, while leveraging the parallel processing capabilities of GPUs to achieve a more immersive and high-quality visual experience. To this end, the total computational cost associated with generating color and occlusion CGHs is evaluated, quantifying the relative contribution of each factor. The results indicate that, under strict occlusion conditions, temporal multiplexing filtering does not significantly impact the overall computational cost of CGH calculation.

## 1. Introduction

Optics provides a framework for describing the propagation of information and energy through electromagnetic waves in the visible spectrum [1]. One of its earliest fields of application was image formation from scenes, and the tools developed for this purpose are well known (e.g., geometrical optics, diffraction theory of light, etc.). The transition from 2D to 3D scene representation introduces greater complexity and entails a shift in the way this information is modeled, stored, and represented. A solution to this challenge is offered by holography, since its discovery by Dennis Gabor in 1948 [2]. Holograms are sophisticated structures that capture both the phase and amplitude of a scene’s wavefront, providing views from all possible perspectives through a given aperture. Holographic visualization techniques allow the inclusion of all features of human vision, including those related to depth [3]: occlusion, ocular accommodation, convergence, and stereopsis.

With the advent of the digital era, such scenes can be defined on a computer and represented through models that integrate 3D geometry, textures, and various appearance attributes, including local and global reflectance properties, and illumination, among others. This gave rise to a discipline within computer science known as computer graphics. In these representations, light is systematically modeled according to the principles of geometrical optics, employing rendering methods that rely on techniques such as projection, rasterization, or ray tracing [4].

Computer-Generated Holograms (CGHs), initially proposed in [5], constitute a branch of holography that models the storage of the wavefront originating from a scene in digital format, and addresses the inverse problem compared to analog holography. A comprehensive overview of the current state of the art in this field can be found in [6].

CGHs implicitly store the behavior of the wavefront emanating from the scene, which entails a significant increase in the amount of information to be managed: CGHs can essentially be considered as a matrix of numbers (ideally complex).

As more features are included in a scene, such as complex illumination, surface textures, and object occlusions, the amount of information grows, requiring a redefinition of how CGHs are computed, since a much higher pixel density becomes necessary.

Various proposals have been developed to address this challenge, aiming to generate scenes with maximum quality while simultaneously minimizing the computation time required for CGH synthesis.

These methodologies can generally be categorized into several techniques. First, point-cloud-based methods track the propagation of discrete points within the scene through space. Second, geometric primitives form the basis of another class of techniques. Additionally, layer-based methods exploit the convolution properties of a wavefront on a plane with a kernel, enabling the propagation of the wave to another plane through transformation operators [7]. Finally, deep learning-based algorithms are currently gaining significant prominence for 2D scenes [8] or 3D scenes using 2D images as layers [9], achieving CGH synthesis times of less than one second once the network has been trained. The synthesis of 3D scenes that include realistic occlusion effects, however, remains an open challenge. Several recent comprehensive reviews [10,11] cover deep learning-based CGH synthesis.

Moreover, polygon-based methods and ray tracing represent notable paradigms in scene rendering. Ray tracing, in particular, is a widely recognized technique used to model illuminated scenes in computer graphics. This method revolves around tracing individual rays of light as they traverse a virtual scene and interact with its surfaces along the way. Through these interactions, ray tracing enables the generation of highly realistic images, making it an indispensable tool in the field of computer graphics.

A comprehensive classification of these techniques can be found in [12], where the algorithms are thoroughly described, including their advantages and limitations. As noted in this reference, no single technique clearly dominates the others, and design decisions must be made accordingly.

CGHs impose significantly higher computational demands compared to images generated through computer graphics. In other words, there is a computational time cost when attempting to provide the spatial perception offered by CGH, in contrast to 2D synthetic images or pseudo-3D obtained through stereoscopy. Although recent advances in computer technology have facilitated the synthesis of holograms on desktop systems, generating a CGH with full parallax, realistic occlusion effects, or high-frequency noise filtering using ray-tracing methods may still require several hours or even days with state-of-the-art hardware.

CGH generation involves finding the right balance between computational cost (time) and the accuracy of the resulting hologram, along with considerations of occlusion, filtering, and color techniques that affect its quality and performance. To better understand the computational load requirements of Computer-Generated Holograms, let us consider a 3D scene discretized with a resolution equivalent to high-definition television (1920×1080 pixels). A spatial light modulator (SLM) serves as the display device for CGHs under appropriate illumination. Assuming a current pixel size of 1 µm, achieving a sufficiently large viewing window (e.g., 27-inch monitors in 16:9 format, corresponding to 65.8 cm in width and 33.6 cm in height) requires approximately $3.45\times 10^{9}$ pixels. Since each pixel of the scene must transmit information to all the pixels of the SLM, the computational effort amounts to approximately $3.15\times 10^{15}$ times that of a single ray. Spatial or temporal multiplexing to generate color entails a threefold increase in computational effort.

The reconstruction of 3D images from CGHs is achieved by recovering the original wavefront through appropriate illumination and propagating the resulting wave through space. To this end, algorithms based on FFT techniques with different propagation kernels are employed [1] (e.g., the angular spectrum method). This image reconstruction has a much lower computational cost and does not represent a significant addition to the total time once the CGH has been computed.

### 1.1. Speckle Denoising

The digital nature of CGHs, the use of coherent light sources, and the resolution limitations imposed by current technology give rise to issues in the generated images related to diffraction and speckle effects.

Speckle is a high-spatial-frequency noise that appears in reconstructed holographic images [13], whether optical or computer-generated. It arises from the random superposition of coherent light waves scattered by the rough surface of an object. Two main approaches are used to eliminate it: optical and digital methods [14]. The former rely on the use of low-coherence light sources (e.g., LEDs) or moving diffusers that introduce a random phase for each exposure, among the most prominent strategies.

The digital approach involves modifying the amplitude and phase pattern calculated for the CGH in various ways [12]. Three main categories can be identified: temporal averaging methods, non-iterative methods, and iterative methods. The first are based on temporal multiplexing of several CGHs (which again increases the computation time), to which statistically independent random phase matrices are added [14,15]. Non-iterative methods rely on the use of spatial filters through matrix operators (e.g., diffusion methods or random phase-free methods). Finally, iterative methods modify the amplitude and phase values of the CGH once its output has been evaluated (e.g., the Gerchberg–Saxton algorithm) [16,17,18], or incorporate realistic images with global illumination effects [19]. However, the use of such feedback techniques does not allow for a deterministic estimation of the computation time required for hologram synthesis.

Currently, artificial intelligence-based approaches are being employed for these tasks. Neural networks are trained with datasets of noisy and noise-free images [20,21,22,23]. Once trained, these networks can remove speckle from a holographic image in real time with high accuracy.

### 1.2. Occlusion Culling for Point Clouds

Occlusion is one of the effects that must be addressed in order to achieve a realistic and immersive viewing experience [12]. Occlusion culling is the process of identifying and discarding points that are hidden from the viewer’s perspective by other points or surfaces, significantly improving rendering efficiency and realism. Techniques involve using projection to sample depth, hierarchical structures, and visibility maps to determine which points are actually visible and should be rendered, especially useful for large indoor scenes and holographic displays where a massive number of points can obscure hidden areas.

Although conceptually straightforward to define, occlusion remains an open challenge in the design and implementation of CGHs due to its high computational cost. Different approaches have been proposed to address this problem [4]: (a) Pseudo-orthographic projection: This technique transforms the point cloud so that the points closest to the hologram plane appear to occupy all positions without gaps, allowing for the straightforward discarding of points located behind them. (b) Hierarchical structures: Data structures such as Bounding Volume Hierarchies (BVHs) [24] enable efficient traversal and visibility determination, allowing the identification of visible parts of the point cloud by examining ancestor nodes. Binary Space Partitioning (BSP) trees are one form of such hierarchical partitioning. BSP trees can be classified as either axis-aligned or polygon-aligned. BSP construction can also be performed recursively by subdividing space into two regions and sorting geometries within these subspaces. A feature of BSP trees is that geometry can be ordered front-to-back along any axis, facilitating position-based visibility checks. Axis-aligned BSP trees are also known as k-d trees. Other approaches used in computer graphics, but not considered in this work, include inverse orthographic projection, visibility mapping (V-Map), and frame-to-frame coherence.

For CGHs calculated from point clouds, several approaches allow their implementation [25], although it is acknowledged that this method is not the most suitable for reproducing true occlusion effects. In [26], a solution based on hogels is presented, which limits the quality of the reconstructed scene. For CGHs computed using a combination of ray-tracing and point-cloud methods [19], several issues are addressed, including occlusion. Timing data reported in this and other studies highlight the demanding computational requirements associated with this problem.

Selecting an appropriate occlusion culling algorithm is important because it improves rendering efficiency, ensures correct depth cues, and reduces computational costs by avoiding unnecessary rendering and calculations. Its application in CGH design is essential for creating accurate and visually convincing holograms, ensuring that points are displayed at the correct depth and that hidden surfaces are properly obscured.

### 1.3. Acceleration Algorithms for CGH Computation

There has been considerable activity focused on improving CPU-, GPU-, and FPGA-based architectures to reduce CGH computation times, following the work of Petz et al., who employed computer graphics hardware to accelerate the calculation process [27]. Owing to the linearity of the diffraction operator, it is particularly well suited for massively parallel implementations and architectures such as GPUs [28] and FPGAs [29,30].

These algorithms are essential for bringing CGH computation closer to real-time conditions. Depending on the initial algorithm, several strategies can be employed to reduce the computational complexity of CGH, including sparsity, reuse of precomputed values, dynamic acceleration, and deep learning-based techniques. The 3D scene is treated as a cloud of discrete points, where for each point the corresponding spherical wave is calculated, and the final hologram results from the superposition of all these waves. For an efficient implementation of these algorithms, the following techniques can be applied: (a) Look-up table (LUT) [31,32]: One of the most efficient approaches for accelerating the computation. Spherical point waves are precomputed and stored in a table. Instead of calculating each wave individually, the algorithm retrieves the corresponding values from the table, which drastically reduces the computation time, although it requires a significant amount of memory for storage. (b) GPU acceleration (CUDA/OpenCL) [28,33]: The inherently parallel nature of point-wave computation makes it well suited for parallel processing. Graphics processing units (GPUs) can evaluate the contributions of hundreds or thousands of points simultaneously, greatly accelerating the process. (c) Deep learning: This technique enables the optimization of non-linear processes [34,35] and can even improve iterative algorithms such as Gerchberg–Saxton [17]. Color CGHs represent a particular case that increases the complexity of the computation [36].

### 1.4. General Approach and Objectives

The main objective of this study is to develop and validate an optimized algorithm for Computer-Generated Holography that addresses speckle noise while simultaneously tackling the issue of occlusion. The research focuses on the implementation of a temporal multiplexing technique and proposes two different approaches to handle occlusion, leveraging the parallel processing architecture of GPUs to achieve a more immersive and high-quality visual experience. While the individual components, GPU acceleration, temporal multiplexing for speckle reduction, occlusion handling, and CGH computation, are well established, their integration in this manner towards a specific objective constitutes a non-trivial research challenge.

The novelty of this work lies in the simultaneous addressing of two key challenges. Most existing studies focus either on computation speed (GPU) to improve occlusion handling or on speckle reduction (multiplexing) in isolation. This article proposes a comprehensive solution that tackles both challenges concurrently, demonstrating how optimizing one aspect can directly benefit the other. A practical approach is thus introduced to advance towards the ultimate goal of real-time holography, which is crucial for the development of future applications in fields such as virtual/augmented reality, data visualization, and medicine.

The article details the algorithm architecture in Section 2, along with the resulting scene images. Section 3 presents quantitative results, including both computation times and image quality under various scenarios. Section 4 analyses the results and images presented in the previous sections, and finally, the conclusions summarize the key findings of this work.

## 2. Materials and Methods

**Figure 1:**
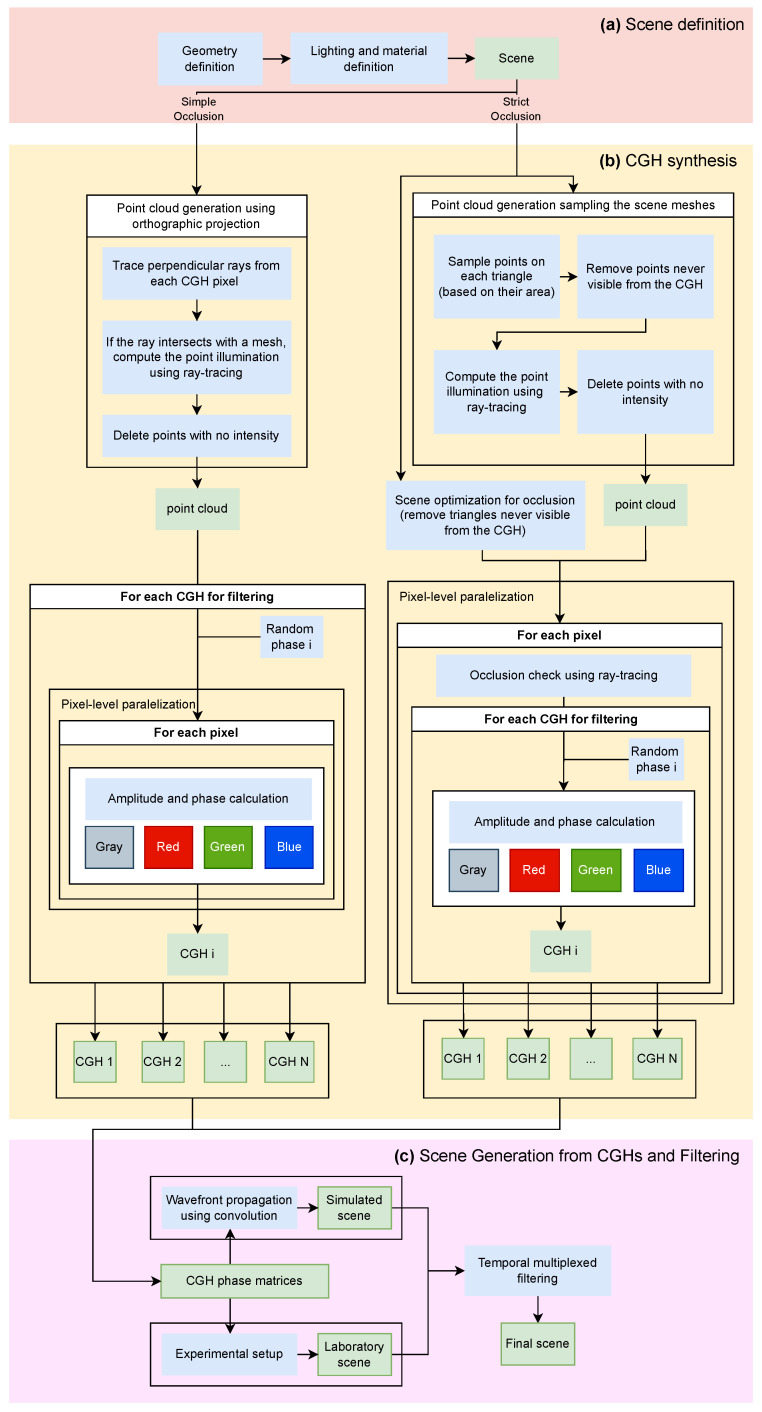
Proposed workflow diagram. (**a**) The scene is generated using computer graphics techniques. (**b**) From the scene, N CGHs are computed, with two possible approaches for occlusion handling (strict or simple). (**c**) Using the N CGHs, the scene is reconstructed either through simulation (using angular spectrum-based propagation techniques) or experimentally in the optics laboratory. Procedures are shown in blue, and results are shown in green.

The proposed workflow is structured into the following stages: first, the definition of the 3D scene (Figure 1a); next, the CGH synthesis (Figure 1b); and finally, the retrieval or reconstruction of the scene (Figure 1c). The first stage relies on computer graphics techniques to recreate a realistic scene. In order to generate realistic scenes from an initial concept, it is necessary to employ and combine several well established techniques and procedures. Starting from an initial idea, computer graphics tools are used to model the scene [4].

CGH synthesis (Figure 1b) essentially involves understanding how the wavefront propagates from the scene to the plane where the hologram is placed. This wavefront can be modeled as a matrix of complex numbers representing the amplitude and phase observed at each elemental unit (pixel) of this plane. In this work, we have chosen to compute the CGH by means of ray tracing and point clouds. The first step is therefore to determine how to obtain the point cloud from the scene while considering occlusion effects. Two alternatives can be distinguished. The first one (referred to here as simple occlusion) has a lower computational cost and is based on the orthogonal projection of the closest points of the scene onto the CGH plane. This information is sufficient for CGH generation. The second alternative (referred to as strict occlusion) generates the point cloud from the geometric coordinates of the scene mesh, which must be preserved in order to decide, for each pixel of the CGH, which points of the scene contribute to its accumulated amplitude and phase.

The occlusion method, same used to construct the point cloud, determines how the CGH value matrix is obtained. In the first case, a random phase matrix is added to generate a specific CGH (CGH(i)). Since the chosen approach involves subsequent speckle filtering by temporal multiplexing, it is necessary to generate N CGHs, each using a different random phase matrix.

On the other hand, in the case of strict occlusion, an additional step is required before computing the CGH. For each pixel, it is necessary to verify whether an element of the point cloud is not occluded by the scene mesh. In such cases, a random phase is added in order to compute its contribution to the amplitude and phase of the evaluated pixel. Once again, if N CGHs are required for subsequent filtering, this procedure must be repeated N times. By applying this process to all pixels of the CGH, a set of N complete CGHs is obtained. CGH synthesis is the most critical part of the work, where GPU acceleration techniques are intensively used to simultaneously address occlusion and speckle filtering.

The reconstruction stage (Figure 1c) is based on understanding how this amplitude and phase information perturbs the propagation of a well-defined wavefront (e.g., a plane wave) and how it evolves through space [37]. The light used to reconstruct this scene, together with the geometric characteristics of currently available CGHs, gives rise to undesired effects that degrade the scene with noise (i.e., speckle) or diffraction when passing through an aperture. To eliminate speckle, N scenes obtained from the N previously computed CGHs are multiplexed. This process can be performed using mathematical models (simulation) or experimentally in the laboratory by transferring the phase information to a physical device, such as a spatial light modulator (SLM). We also present simulations of color scenes, resulting from the multiplexing of CGHs calculated for three different wavelengths.

In the remainder of this section, we describe each of these steps in detail, emphasizing their most relevant aspects.

### 2.1. Scene Definition: Computer Graphics and Lighting Modeling

To generate a photorealistic scene geometry, appearance (textures), material behavior under illumination [38,39], light sources, and other relevant factors must be taken into account. All this information is specified in a scene file, which can be visualized (using a modeled camera) or used as input for the next step: the computation of the hologram or CGH. This stage corresponds to box (a) in Figure 1.

**Figure 2:**
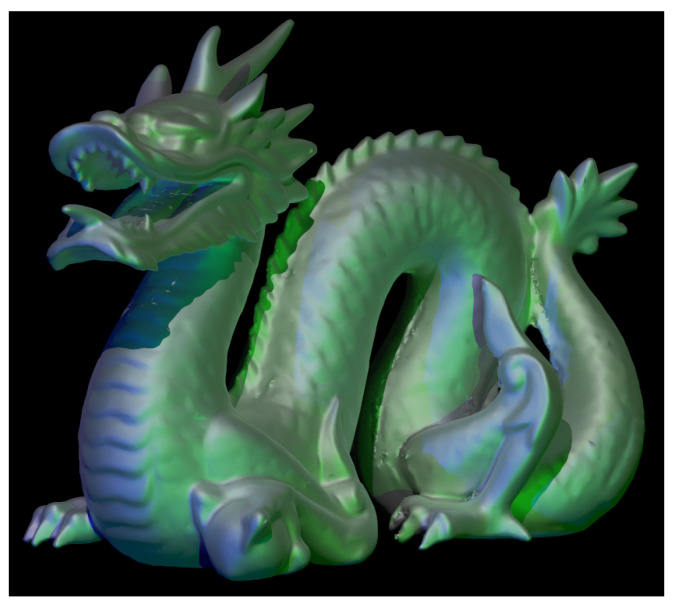
Three-dimensional scene detail. Stanford dragon illuminated by three point light sources.

Taking all of these factors into account, a scene image is generated using ray tracing with 128 samples per pixel in 33 s (Figure 2), using the C++ language with gcc-14 compiler running on a Intel i9-9900K CPU, Santa Clara, CA, USA. The scene contains a well known object in computer graphics, the Stanford dragon, because it has a high polygon count. The dragon is illuminated by three point light sources (blue, white, and green) placed on the left, top, and right, respectively. The full image has a resolution of 1920×1080 px and 15.36×8.64 mm, which matches both the resolution and the size of the SLM that will later be used in the laboratory [40].

### 2.2. CGH Synthesis

In the present work, a hybrid technique combining point clouds (based on discretizing the scene into samples) and ray tracing (to model light transport and occlusion) is employed [19,41,42,43]. This approach involves generating a point cloud from a scene and computing its illumination using ray tracing, achieving realistic lighting while maintaining coherence (the same point is used across all pixels). We have focused on the ray-tracing technique because each method has its own advantages and limitations. We consider the main advantage of ray tracing over layer-based methods to be its ability to accurately handle strict occlusion effects. Reference is made to Figure 1b to support the subsequent subsections.

#### 2.2.1. Occlusion Culling for Points Cloud

The main challenge in CGH synthesis lies in finding a balance between rendering the scene as realistically as possible and achieving this goal within an acceptable computation time. Realistic rendering involves textures, surface structural properties, non-uniform illumination, and occlusions, all of which are necessary to enhance realism and immerse the observer.

**Figure 3:**
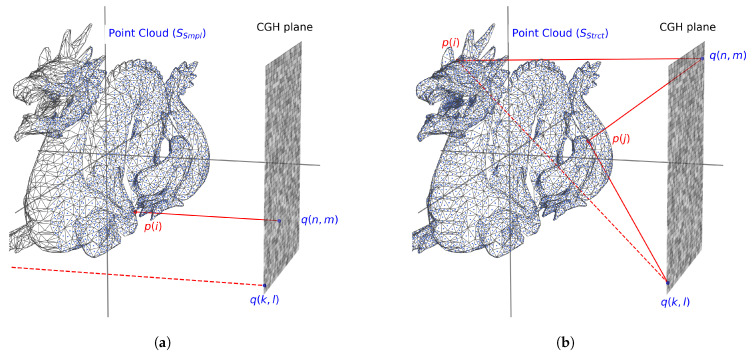
Basic schemes for the occlusion implemented in this work (Figure 1b). (**a**) Simple occlusion: for scenes with suitable geometry, the point cloud $S_{Smpl}$ is obtained with an orthographic projection from the CGH pixels to the scene. (**b**) Strict occlusion: the point cloud $S_{Strct}$ is obtained by sampling the mesh. During CGH generation, the visibility of each point is checked for each CGH pixel.

One of the main characteristics of the point-cloud technique is algorithmic speed, albeit at the cost of not addressing occlusion or realistic lighting. Ray tracing complements these shortcomings by providing solutions for both occlusion and realistic illumination. Our approach includes two levels of occlusion culling, both of them with a different way of generating a point cloud and computing the hologram (Figure 3): simple and strict occlusion.

##### Simple Occlusion

Depending on the size of the CGH, the scene, and the distance between them, a point cloud $S_{Smpl}$ is generated from a pseudo-orthographic projection of rays (emanating from each pixel of the CGH), with the illumination of each intersection point with the scene geometry computed via ray tracing (Figure 3a). Points with zero intensity can be discarded, as they do not contribute to the CGH. In this way, it can be assumed that all points are visible to all pixels of the CGH. So, to compute the hologram, each point contributes to each pixel of the CGH. This approach allows for the accurate inclusion of the scene depth, although it does not account for strict parallax effects. Nevertheless, it can be useful in scenes where parallax is not the dominant effect (for instance, when the scene size is comparable to that of the CGH).

This allows leveraging the speed of the point-cloud technique while approximating occlusion, provided that the scene is located at a distance much greater than the size of the SLM. A pseudocode representation of the algorithm is provided in Algorithm 1. In this case, each CGH used for subsequent filtering is computed sequentially, independent of the others, as the computational bottleneck is the point-to-pixel propagation, which is computed using pixel-level parallelization.
**Algorithm 1** Pseudocode used to describe simple occlusion (Figure 3a).
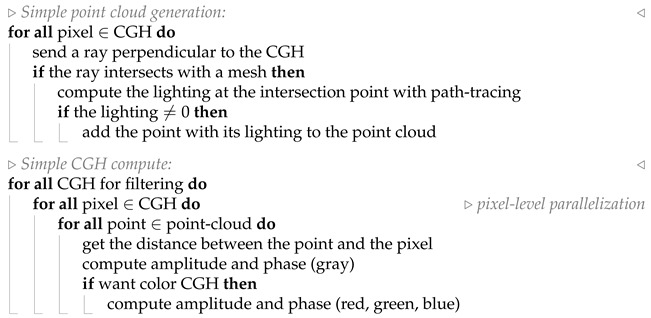


So, assuming that point-cloud is precomputed, the only factors that affect computational cost are the point-cloud size, the number of CGHs (*N*) for filtering and the CGH’s resolution.

The point cloud $S_{Smpl}$ is obtained with an orthographic projection from the CGH pixels to the scene. Rays that do not intersect (like the one from q(k,l)) the scene are ignored (dashed line). We then assume that all points contribute to the computation of each pixel. The density of the point cloud depends on the size of the CGH (which corresponds to the size of the SLM used for the reconstruction) and on the number of points it contains, namely 15.36×8.64 mm and 1920×1080 px, respectively.

##### Strict Occlusion

If the geometry of the problem does not allow for simple occlusion, or full occlusion is desired, it is necessary to compute the occlusion of each point for every pixel of the CGH. The computational cost of this process is very high, as all elements of the scene must be considered. For this approach, the point cloud must be generated differently from the simple occlusion approach (see Figure 3b). Instead of casting rays from the CGH, a point cloud $S_{Strct}$ is generated by sampling points on the surfaces of triangle meshes. As an optimization, points are removed if the angle between the normal of the triangle and the direction of the CGH is less than ($\frac{\pi }{2}-\Delta \alpha$), where Δα depends on the relative position of the CGH pixel with respect to the point. The illumination of each point is computed via ray tracing and points with zero intensity can be discarded. If the point is visible (solid line), it contributes to the pixel; if it is not visible (dashed line), it does not contribute. In this case, point p(i) contributes only to q(n,m) and point p(j) contributes to both q(n,m) and q(h,l).

The point cloud $S_{Strct}$ is defined from the vertices of the scene mesh. For triangles with an area larger than the threshold specified by the designer, additional points are randomly selected within the triangle in order to achieve a sufficiently high point density to convey a sense of continuity across the surfaces.

To compute the CGH, each pixel determines occlusion for each point by tracing a ray from the pixel to the point and checking whether it intersects with the scene geometry before reaching the point. If the ray intersects, meaning there is geometry occluding the point, it does not contribute to the CGH pixel (Figure 3b).

The workflow diagram (Figure 1b) and the pseudocode (Algorithm 2) briefly illustrate that the phase and amplitude of each pixel are computed at the same time for the *N* CGHs to be used in a subsequent filtering process, as they can share the pixel-to-point occlusion check, wich is the computational bottleneck.
**Algorithm 2** Pseudocode used to describe strict occlusion (Figure 3b). The most relevant lines are underlined for emphasis
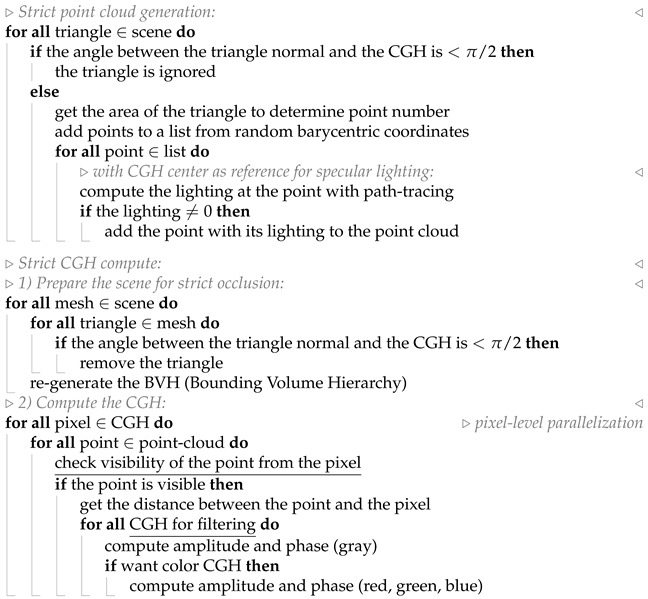


So, for strict occlusion, the factors that affect the computational cost are the number of points selected from the cloud, the number of CGHs (*N*) used for filtering, the CGH’s resolution, and the scene geometry (i.e., the number of objects and triangles and their positions).

#### 2.2.2. Amplitude and Phase Propagation: Grayscale and Color CGH

Once a decision has been made regarding the occlusion of the scene points, the amplitude and phase reaching each element of the CGH must be computed. This is achieved through the coherent superposition of the contributions from sampled points [1].

(1) $$U\left(\vec{q_{j}}\right)=\sum_{S}U\left(\vec{p_{i}}\to \vec{q_{j}}\right)=\sum_{S}U\left({\vec{p}}_{i}\right)e^{j(\vec{k}\cdot \vec{r_{ij}})}$$

where $\vec{q_{j}}$ are the coordinates of a CGH pixel, $\vec{p_{i}}$ those of a scene point, and *U* denotes the complex amplitude propagating in space. Here, $\vec{r_{ij}}=\vec{q_{j}}-\vec{p_{i}}$, and *S* is the set of samples obtained from the scene.

In order to compare the calculated results with those obtained in an optical laboratory, the computed phase is stored in an image format so that it can later be displayed on an SLM. The chosen format is PNG with 8 bit uniform linear quantization for each channel (red, green, blue, and grayscale).

**Figure 4:**
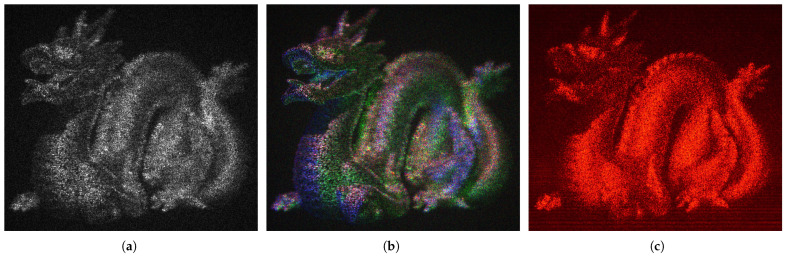
Scene reconstruction from grayscale CGH computation (**a**), color CGH computation (**b**), and laboratory capture (**c**).

The result of this process is shown in Figure 4a for a CGH computed from grayscale levels. For this CGH, a HeNe laser wavelength (λ=0.633 µm) is used in order to compare the simulation results with those obtained in an optical laboratory.

Figure 4b shows the result of computing three CGHs at different wavelengths to produce a final color image ($\lambda_{r}=0.633$ µm, $\lambda_{g}=0.532$ µm, $\lambda_{b}=0.442$ µm), corresponding respectively to HeNe, DPSS, and He-Cd lasers [44]. The color image is obtained by temporal multiplexing of these three CGHs, illuminated with the same wavelengths used for phase calculation.

These figures are the result of a simulation in which a plane electromagnetic wave with λ=0.633 µm is propagated (for the case of Figure 4a) and perturbed by the phase variations introduced by the CGH computed from the grayscale scene. The resulting field is observed at one of the object planes (coinciding with the dragon’s head), which explains why the tail appears out of focus. Similarly, the color scene is obtained by repeating the simulation process for the three plane waves used in the recording and temporally multiplexing the results.

Figure 4c shows the image obtained experimentally in the laboratory from the CGH used in Figure 4a. In all cases, the speckle effect is observed, which is typical in diffractive optics when using coherent illumination.

#### 2.2.3. GPU Acceleration

To bring CGH generation via ray tracing closer to real-time performance, a key technique is GPU (graphics processing unit) acceleration. Ray tracing is computationally intensive, and GPUs, designed for massively parallel processing, are well suited for this type of task.

Rather than computing the hologram on a CPU, which contains dozens of general-purpose cores, a GPU contains thousands of cores optimized for parallel programming. This is particularly advantageous for point-based CGH methods, where the contribution of each point must be calculated independently for every hologram pixel.

Other features that make GPUs suitable for this problem include the SIMT [45] (Single Instruction, Multiple Threads) execution model, which allows a single instruction to be applied simultaneously across multiple threads, and the availability of high-speed memory (VRAM), which enables large data transfers at greater speed.

In general, GPUs have more performance for 32 bit (float) than 64 bit (double) floating point operations. Using float variables can introduce phase calculation errors in cumulative processes or when distances between scene points and the CGH are large relative to the recording wavelength. Using double-precision variables solves this issue, but significantly reduces GPU execution speed. Our implementation uses the latter.

The main challenges in implementing the proposed algorithm on GPUs had included efficiently managing memory, enabling CPU-GPU communication, and adapting the algorithm to the specific instruction set of the GPU. For this purpose, the CUDA framework [46] had been employed, as an NVIDIA GPU was available and it provided fine-grained control, along with the use of C++ (gcc-14 compiler) as the programming language. It had also been necessary to modify the data structures from the CPU implementation to comply with the GPU memory model, particularly the scene representations (meshes, materials, illumination) and the Bounding Volume Hierarchies (BVHs) used to accelerate intersection calculations.

In order to parallelize in both the CPU and the GPU, a thread is launched for each pixel of the CGH, as their computations are independent of each other. The results are stored in an array of pixels, *N* arrays in the case of strict occlusion, one for each of the CGHs computed simultaneously. The CPU implementation uses C++ as the programming language and OpenMP [47] as the parallelization library.

### 2.3. Scene Generation from CGHs and Filtering

Once the CGHs have been computed, the scene information can be reconstructed either through simulation or in the laboratory, as thoroughly described in [42]. Among the different filtering methods discussed in Section 1.1, we have chosen temporal multiplexing, based on previous studies, since its computational cost can be reliably predicted.

Temporal averaging techniques are based on the temporal multiplexing of CGHs, which are computed using statistically independent random phases, and have been shown to be effective in speckle reduction. Figure 1c illustrates the two options proposed in this work. From the previously computed *N* CGHs, either by simple or strict occlusion, an angular spectrum propagation algorithm [1] is employed to determine the value of a plane wavefront at one of the planes corresponding to the location of the scene. Alternatively, the scene can be reconstructed experimentally in the laboratory using a PLUTO 2 phase SLM [40], a HeNe laser, and the required optics to generate a plane light beam. The scene is then recorded with a lensless digital camera. A basic schematic is provided in Appendix A.

For the simulated scenes, a CGH encoded in an image file (PNG) with a range of [0, 255] is used. For the scenes obtained in the laboratory, it is necessary to perform an SLM calibration process to optimize its performance, as described in [48]. Consequently, the calibrated CGH phase is stored as an image file in PNG format, with values in the range [4, 121].

**Figure 5:**
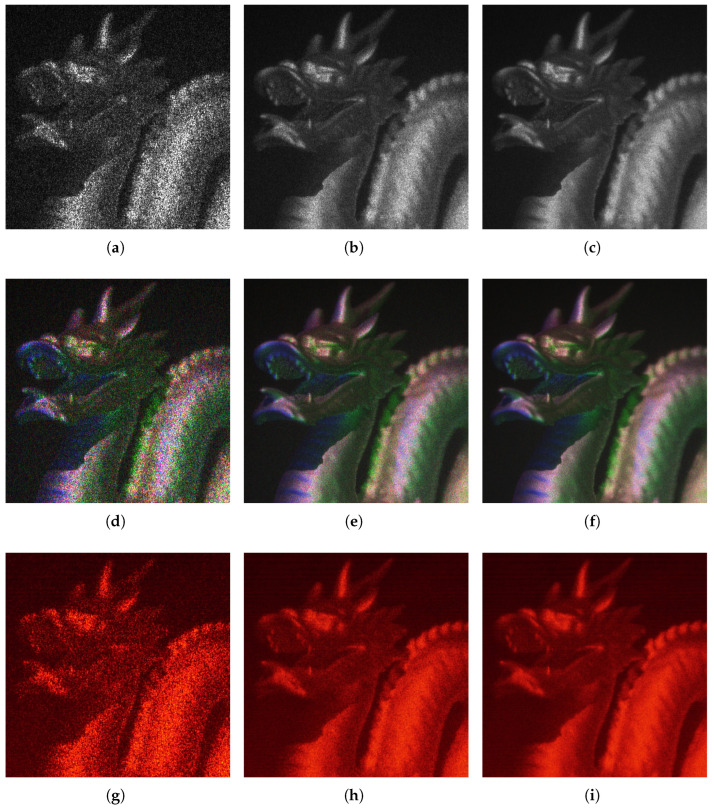
Detail of the test scene as a function of the number of CGHs used for filtering (*N*). The first row shows grayscale CGH simulation results for N=1 (**a**), N=10 (**b**), and N=25 (**c**). The second row presents color simulation results for N=1 (**d**), N=10 (**e**), and N=25 (**f**). The third row shows laboratory results for N=1 (**g**), N=10 (**h**), and N=25 (**i**).

Figure 5 presents details of the reconstructed scenes corresponding to the CGHs used for the final filtering. Both grayscale and color simulations are shown, along with laboratory-acquired images for the grayscale case. On the downside, these techniques are computationally very expensive, which limits their use in real-time applications. For completeness, Appendix B presents the full scene images for N=25.

To obtain quantitative measures of image improvement, the Pearson Correlation Coefficient (CC) [49] has been used to compare the reconstructed images *I* with the original scene *R*, defined as

(2) $$CC=\frac{\sum_{N,M}^{p,q}\left(I_{p,q}-\bar{I}\right)\left(R_{p,q}-\bar{R}\right)}{\sqrt{\left(\sum_{N,M}^{p,q}{\left(I_{p,q}-\bar{I}\right)}^{2}\right)(\sum_{N,M}^{p,q}{\left(R_{p,q}-\bar{R}\right)}^{2}})}$$

where *I* and *R* denote two images being compared, *p* and *q* represent pixel coordinates, $\bar{I}$ and $\bar{R}$ are the mean values of images *I* and *R*, respectively, and $I_{p,q}$ signifies the pixels at p,q in the image under comparison with the reference image $R_{p,q}$.

The Correlation Coefficient (CC) ranges from −1 to 1, where −1 indicates anti-correlation, 0 indicates no correlation between the images, and 1 corresponds to a perfect match. This metric allows evaluating the differences between the reconstructed images and the original scene (Figure 2) as a function of the number of images *N* used for speckle reduction.

**Figure 6:**
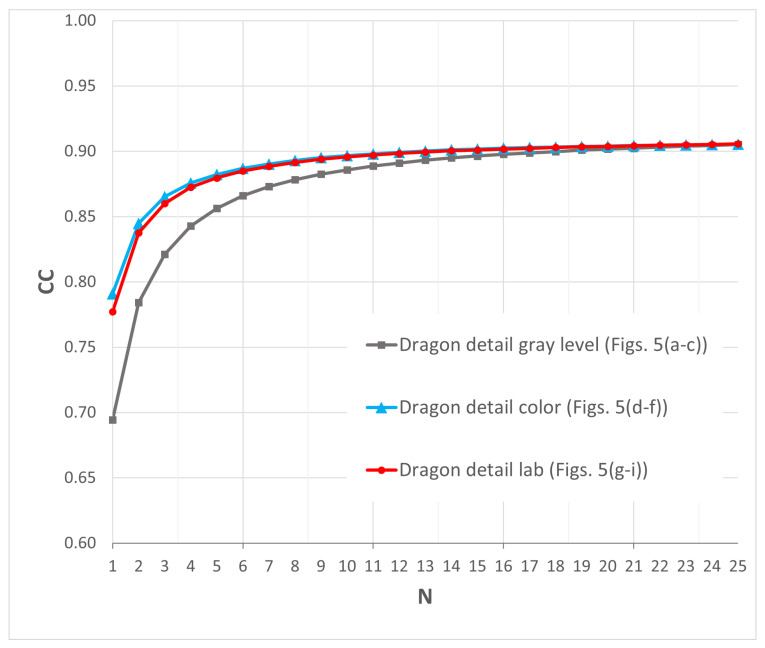
Correlation Coefficient (CC) of the reconstructed scenes (Figure 5) vs. *N* (CGHs used for filtering). Figure 2 is used as the reference image.

Figure 6 shows the behavior of the Correlation Coefficient (CC) as the number of images used for filtering increases, both for CGHs computed in grayscale (gray lines), and for those computed in color (blue lines) and lab scenes (red lines). In this plot, the CC exhibits a clearly asymptotic behavior, allowing designers to select the number of CGHs needed to improve an image without compromising the final quality. Several factors prevent the CC from reaching a value of one. The most important is related to how the reconstructed scenes are captured: to highlight the depth effect of the CGHs, only a single object plane (coinciding with the dragon’s head) is fully in focus. As one moves away from this plane, a defocusing effect appears, which affects the image used for comparison with the original scene.

The asymptotic behavior of the CC is clearly observed. In fact, for N>25, the CC hardly varies, which is why we do not present these data in this graph. Additionally, a difference in behavior is observed for very low values of *N* (i.e., N=1,2 …), which we attribute to the behavior of the speckle in the three evaluated scenarios.

## 3. Results

The scenes were generated taking into account the following variables: selected points, use of CPU and GPU, application of two methods of occlusion (strict versus simple), color versus grayscale scenes, and, finally, temporal multiplexing filtering using *N* CGHs with independent random phases.

The hardware used comprised an Intel i9-9900K CPU, Santa Clara, CA, USA (16 threads) [50] and an NVIDIA GeForce RTX 2060 GPU (TMSC, Taiwan) (1920 cores) [51]. The proposed algorithm illustrated in Figure 1 was tested under these various conditions.

### 3.1. Computational Cost per Scene Point

To compare the performance, the average computational cost of propagating amplitude and phase from a single scene point to all pixels of the CGH was measured, for both grayscale and color CGHs, and under simple and strict occlusion conditions.

Table 1 and Table 2 show the results when the scene is modeled using a 10,000 point cloud. These data show the time consumed using the GPU for N=1, N=10 and N=100 CGHs. They also show the ratio between execution times, using the GPU N=1 value as the reference.

We do not explicitly address variations in computation time arising from factors external to the workflow (e.g., CPU/GPU usage by other tasks), as such variations are minimal. Table 1, Table 2 and Table 3 report the average computation times obtained from three independent tests. These variations do not affect the overall discussion or the conclusions presented in this work.

For simple occlusion condition, Table 1 shows that increasing *N* on the GPU leads to a near linear increase in computation time. Using color takes 3 times more than grayscale, as it has to compute three CGHs for three different wavelengths.

Table 2 presents the results obtained under strict occlusion conditions. The GPU implementation using the improved algorithm proposed in this work does not scale linearly, as the computational bottleneck lies in the occlusion check; the same verification process is applied to all CGHs for filtering. This level of performance could not be achieved by simply combining approaches that handle both problems independently, as evidenced by the without improvement columns in the same table. The performance difference compared to the improved algorithm reaches 9.99 and 9.76 times for N=10, and 83.7 and 60.04 times for N=100, for grayscale and color CGHs, respectively.

This table shows how the relationship between the computation time required for occlusion and the time required for point-to-pixel propagation changes completely, and only in the case of calculating a color hologram with N=100 does the effect of the latter computation become noticeable. The geometry also affects the time. If a point is occluded, it does not compute the point-to-pixel propagation.

The results obtained using the CPU, which cannot be directly compared with the GPU, are the following: CPU N=1, simple occlusion, grayscale: 6.81 ms, color: 19.37 ms; CPU N=1, strict occlusion, grayscale: 574.68 ms, color 593.56 ms. It should be noted that computing 10 CGHs with a CPU using simple occlusion implies multiplying the computation time by a factor of 10, and similarly by 100, or in general by *N*.

### 3.2. Total Computational Cost per Scene

Table 3 shows the computational cost per scene, taking into account the number of selected points in the scene, the occlusion algorithms, and the number *N* of CGHs used for temporal multiplexing filtering. The results again show a linear behavior for simple occlusion (both with respect to the number of selected points and the number of CGHs) and a linear behavior only with respect to the number of selected points in the case of strict occlusion. In this case, increasing *N* does not have a significant effect on the computational cost.

The number of points in the last column differs for both occlusion methods because the perceptual image quality at the same point count differs. The point clouds are obtained differently: with the simple occlusion, the points are sampled with the CGH position in mind and all of them contribute to the CGH, 371 K is the number of rays that intersect with the scene for a 1920×1080 resolution CGH. However, with strict occlusion, the points are sampled based on the triangles of the meshes and it is necessary to have higher point-cloud density to obtain similar perceptual results.

## 4. Discussion

This work is framed within the context that, at present, potential advances in CGH synthesis aimed at producing scenes with high visual quality are limited by SLM fabrication technology. Two main challenges must be addressed: reducing the pixel size and increasing the overall size of the SLM. In both cases, the CGH matrix to be computed becomes significantly larger, resulting in a higher computational cost.

In this context, occlusion and filtering are two essential tasks for achieving realistic effects when reproducing scenes stored in CGHs; however, they are also the most demanding in terms of computational resource consumption. Previous research has devoted considerable effort to reducing computation times for each of these challenges individually.

When the decision is made to reduce speckle noise through temporal multiplexing, it is necessary to generate N scenes, whose computational cost depends on the type of occlusion employed. If an orthonormal projection-based occlusion is used, the cost of generating a filtered scene from N individual CGHs increases linearly with N. Conversely, if the occlusion strict is computed by evaluating which scene points affect each pixel of the CGH individually, the main cost is essentially this calculation itself, and generating N CGHs requires only slightly more time.

The data presented in this work demonstrate that, in the case of strict occlusion, computation times can be significantly reduced by addressing both problems from a global perspective and by exploiting the parallel processing architecture provided by modern GPUs.

The specific architecture employed also influences overall performance. Nonetheless, the hardware used is not a determining factor in the results reported here. The proposed algorithm can be implemented on more advanced GPUs. In particular, we expect a significant improvement when using GPUs with high 64 bit floating point (double) performance.

The choice of floating point precision (float (32 bit) or double) is also critical, and a balance must be sought between generation speed and the tolerable phase calculation errors. GPU architectures impose stringent restrictions on the precisions that can be efficiently employed. In this work, double precision variables have been used, as they provide adequate quantization of the propagated ray phases; however, this choice slows down computation on GPUs, which perform significantly worse with double precision than with float precision.

In summary, the contribution of this article does not lie in a single element, but rather in the holistic integration of mature solutions to provide an innovative and practical response to a persistent problem in holography.

The approach presented in this work is compatible with the use of optimization algorithms, specifically those based on Monte Carlo techniques [42,43], which allow computational costs to be reduced while controlling the quality of the final scene. To accelerate the CPU, techniques like SIMD programming, branchless programming, and cache-aware programming could be used. To accelerate the GPU, hardware-accelerated ray tracing will benefit the point-cloud generation as well as the strict occlusion visibility check.

## 5. Conclusions

We present a complete workflow that, starting from an initial design, enables the storage of a 3D scene with speckle reduction through temporal multiplexing of *N* CGHs.

Two occlusion techniques are employed, referred to as simple and strict, which determine the computation time required for each hologram. To optimize this computational cost, we propose incorporating, within the strict occlusion process, the calculation of the amplitudes and phases necessary for filtering.

Simulated results are provided for both grayscale and color scenes. Additionally, grayscale scenes have been experimentally reproduced in the laboratory.

The computation times are measured in each case, both per CGH pixel and for the overall scene calculation. The results indicate that the proposed approach significantly reduces this value compared to an individual computation of each CGH.

To assess the improvement in image quality, the Correlation Coefficient is used as a metric. The results show an asymptotic behavior from N=15, allowing the designer to determine the final number of CGHs required to generate the scene while limiting computation time.

The designer can choose between simple and strict occlusion to achieve a realistic 3D effect: simple occlusion is effective when the occlusion effect is not dominant compared to the depth effect, for example, when the scene is located far from the CGH plane and its lateral dimensions are comparable. In this case, the time measurements reveal a clear reduction. When strict occlusion is required, our proposal ensures that the generation of *N* CGHs does not significantly affect the overall computation.

### Future Work

Although the approach presented in this work reduces computational costs, there remains room for improvement by incorporating optimization techniques to decrease the number of rays required to synthesize a CGH. In this regard, efforts are underway to integrate Monte Carlo-based algorithms.

## Figures and Tables

**Table 1:** Computational cost using simple occlusion per point (ms). *N* denotes the number of CGHs computed for a subsequent temporal multiplexing filtering process. Ratio data are computed based on the cost of generating one grayscale CGH using the GPU as the reference unit ^1^.

GPU × *N*	Grayscale CGH		Color CGH	
	Time	Ratio	Time	Ratio
GPU × 1	1.16 ^1^	1.00	2.99	2.58
GPU × 10	11.57	9.97	29.92	25.79
GPU × 100	115.69	99.73	299.15	257.89

^1^ Time used as reference for ratio data.

**Table 2:** Computational cost under strict occlusion per point (ms). *N* denotes the number of CGHs computed for the subsequent temporal multiplexing filtering process. The table includes data obtained both with and without the improved algorithm. The ratio values are calculated using the cost of generating one grayscale CGH with the improved GPU implementation as the reference unit ^2^.

GPU × *N*	Grayscale CGH				Color CGH			
	*w/o* ^1^		*w/*		*w/o*		*w/*	
	t (ms)	*Ratio*	t (ms)	*Ratio*	t (ms)	*Ratio*	t (ms)	*Ratio*
GPU × 1	123.60	1.0	123.60 ^2^	1.00	120.75	0.98	120.75	0.98
GPU × 10	1248.70	10.10	124.90	1.01	1219.33	9.87	130.76	1.06
GPU × 100	12,423.43	100.51	148.41	1.20	12,134.22	98.17	202.11	1.64

^1^*w/o*: without improvement. *w/*: with improvement. ^2^ Time used as reference for ratio data.

**Table 3:** Computation time per scene (*s*) using the GPU as a function of the number of CGHs computed for filtering (*N*), occlusion method (simple or strict), and the number of selected points in the point cloud for color CGH synthesis.

*N*	Simple Occ.				Strict Occ.			
	1 K	10 K	100 K	371 K	1 K	10 K	100 K	800 K
1	3.18	29.91	311.67	1135.5	121.36	1207.51	13,173.57	100,817.3
10	31.76	299.01	3116.08	11,355.9	128.84	1307.59	13,873.51	107,174.1
100	317.54	2989.47	31,154.53	113,552.4	197.52	2021.14	21,835.72	166,885.2

**Figure A1:**
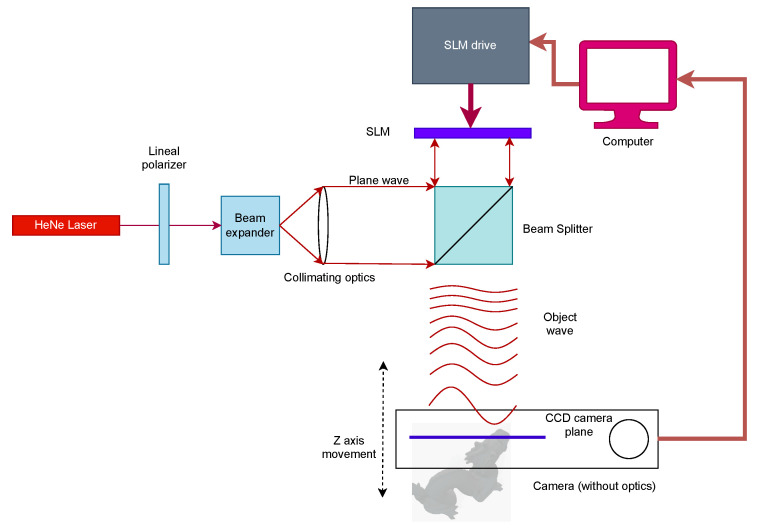
Experimental setup for recovering 3D scene.

**Figure A2:**
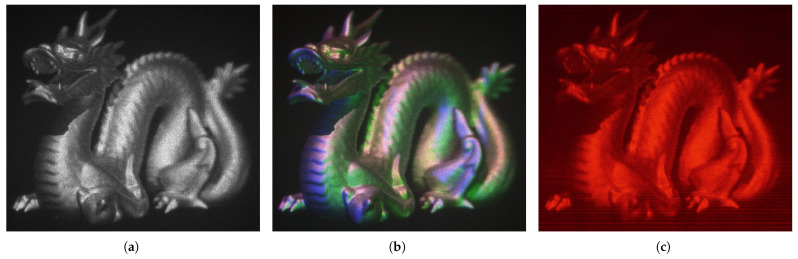
Scene reconstruction after filtering obtained from (**a**) grayscale CGH computation, (**b**) color CGH computation, and (**c**) laboratory capture.

## Data Availability

The data presented in this study are available on request from the corresponding author.

## Appendix A

Schematic of the experimental setup for reconstructing a scene from *N* CGHs (Figure A1). This configuration is commonly employed in the optics laboratory for scene reconstruction from CGHs projected through a commercial SLM (in our case, a PLUTO 2 phase SLM). The distance between the SLM and the camera is 300 mm (and the size of the scene is 6×5.44×6 mm).

The camera (Sony α6000. Sony Group Corporation, Bangkok, Thailand with a resolution of 6000×4000 and 3.88 µm pixel pitch) sequentially records the *N* CGHs, and these are later combined by averaging in order to improve the quality of the reconstructed scene. This averaging can be carried out in two ways: either optically, by using a sufficiently long exposure time so that multiple CGHs contribute to a single image, or digitally, by first capturing the individual scenes corresponding to each CGH and then performing the averaging on a computer.

## Appendix B

Scenes obtained after the filtering process for N=25, both in simulation and in the laboratory. Since the angular spectrum algorithm provides the wavefront value at a specific plane, the rest of the scene appears out of focus. The same effect is observed in the experimental setup. In the presented images, it can be seen that the focal plane coincides with the dragon’s head.
